# Investigation of the degree of organisational influence on patient experience scores in acute medical admission units in all acute hospitals in England using multilevel hierarchical regression modelling

**DOI:** 10.1136/bmjopen-2016-012133

**Published:** 2017-01-18

**Authors:** Paul Sullivan, Derek Bell

**Affiliations:** NIHR CLAHRC for Northwest London, Imperial College, London, UK

**Keywords:** GENERAL MEDICINE (see Internal Medicine)

## Abstract

**Objectives:**

Previous studies found that hospital and specialty have limited influence on patient experience scores, and patient level factors are more important. This could be due to heterogeneity of experience delivery across subunits within organisations. We aimed to determine whether organisation level factors have greater impact if scores for the same subspecialty microsystem are analysed in each hospital.

**Setting:**

Acute medical admission units in all NHS Acute Trusts in England.

**Participants:**

We analysed patient experience data from the English Adult Inpatient Survey which is administered to 850 patients annually in each acute NHS Trusts in England. We selected all 8753 patients who returned the survey and who were emergency medical admissions and stayed in their admission unit for 1–2 nights, so as to isolate the experience delivered during the acute admission process.

**Primary and secondary outcome measures:**

We used multilevel logistic regression to determine the apportioned influence of host organisation and of organisation level factors (size and teaching status), and patient level factors (demographics, presence of long-term conditions and disabilities). We selected ‘being treated with respect and dignity’ and ‘pain control’ as primary outcome parameters. Other Picker Domain question scores were analysed as secondary parameters.

**Results:**

The proportion of overall variance attributable at organisational level was small; 0.5% (NS) for respect and dignity, 0.4% (NS) for pain control. Long-standing conditions and consequent disabilities were associated with low scores. Other item scores also showed that most influence was from patient level factors.

**Conclusions:**

When a single microsystem, the acute medical admission process, is isolated, variance in experience scores is mainly explainable by patient level factors with limited organisational level influence. This has implications for the use of generic patient experience surveys for comparison between Trusts and should prompt further research to explore if more discriminant surveys can be developed.

**Strengths and limitations of this study**

- This study analysed patient experience data for all acute hospitals in an entire country.
- The data included self-reported medical conditions and details of degree of disability that provide comprehensive patient level descriptors.
- The data are from a national survey administered with standardised methodology in all hospitals.
- It is possible that questions other than those in the Adult Inpatient Survey (AIPS) covering different aspects of care might reveal stronger hospital level effects. Further research is needed to determine whether more discriminant surveys can be designed around patient subpopulations.
- The AIPS questions were validated across the whole inpatient population and may neglect aspects of care that acute medical patients would prioritise.

## Introduction

Patient experience is regarded as a key aspect of quality at macro (policy), meso (hospital management) and micro (clinical staff interacting with patients) levels.^1^ Surveys are used in several national programmes to collect data on patient experience and to compare hospitals and healthcare organisations, with the implicit assumption that interorganisational differences in scores reflect meaningful differences in local care delivery.^2^ The notion that there are good and bad hospitals is underscored by the current trend of public reporting of performance data, the rising number of rating and comparison websites and the publicity surrounding events such as those at the Mid Staffordshire NHS Trust.^3–5^ However, previous studies that have used multilevel regression models to apportion overall variance of hospital inpatient experience scores between hierarchical levels have shown that the majority of the variation seen can be accounted for by factors outside organisations' corporate control, including patient demographics, presence of long-term conditions, length of stay and urgency of admission.^6^ ^7^ When specialty is included as a level, variation in experience score is similarly largely attributable to patient level factors.^8^ ^9^

One possible explanation for the relatively small organisational contribution to overall variation in models may be that quality of experience is heterogeneous across the range of subunits that make up an organisation. If so averaging scores across a whole hospital, for example, may mask examples of good and bad practice and create a regression to the mean effect. Similarly, whole specialties may be heterogeneous, consisting of subteams each with its own leadership and style, and often with patients in a number of different locations within an organisation. We aimed to determine whether organisational contribution to variation in experience score is more apparent at the level of a standard reproducible microsystem, which performs the same role in a large number of healthcare provider organisations: the acute medical admission process. In England, this process is delivered in a single location in each hospital, the acute medical unit (AMU), which receives and cares for emergency general internal medicine admissions referred from the emergency department and from community general practitioners (primary care providers). AMUs provide care for the initial part of a hospital stay (typically <48 hours) prior to transfer to a general or specialty ward or in the case of shorter admissions, for the entire stay.^10^ ^11^ While the group of patients admitted into AMUs have a range of different presenting symptoms and medical conditions, they all go through a standardised linear process—nursing assessment, much of which is nationally mandated, medical ‘clerking’ by a trainee doctor using a stereotyped format, diagnostics such as X-rays, scans and blood tests, then review by a senior clinician and communication regarding their illness. The group of patients included in this study were discharged directly from the AMU, and their entire encounter would be largely limited to going through the steps above, with or without therapeutic intervention, followed by discharge information and instructions. The acute medical admission process could be regarded as more homogeneous than a whole hospital specialty, since specialties deal with patients undergoing a range of different types of clinical processes.

We used data from the English national Adult Inpatient Survey (AIPS). If the AIPS is able to demonstrate a meaningful degree of variation in experience during the acute admission process attributable to Trust level, it would be attractive for it to be administered in larger numbers in AMUs in a national scheme to drive improvement of the acute admission process through comparison and benchmarking. This is an important area for study as acute medical patients represent a substantial proportion of admissions to hospital and report worse experience than other inpatient groups.^12^

## Methods

We used data from the AIPS for years 2008, 2009 and 2010 for entire English National Health Service, which provides virtually all emergency care for a population of 53 million.^13^ This period was selected because 2010 was the latest year that included information that can be used to identify acute medical admissions. Data were retrieved from the National Data Archive.^14–16^ We did not seek ethical approval as all data were collected routinely for other purposes and were available in the public domain. The AIPS has been shown to retain validity when analysed at suborganisational levels.^17^ The survey is administered to a sample of 850 patients annually in each of the 167 National Health Service Acute Provider Trusts in England using a standardised methodology (see box 1 for details). We included all Acute Trusts that receive unselected emergency medical admissions. We included acute patients coded to medicine who were at least 16 years old and had length of stay of one or two nights, did not move from their initial inpatient location and were not admitted to intensive care, high dependency or coronary care units.

Box 1 **The English Adult Inpatient Survey**

- Administered annually since 2002.^22^
- Standardised methodology in all English NHS Acute Provider Trusts.
- Questionnaires are posted to the first 850 unselected consecutive inpatients discharged after a predetermined date in each acute provider trust.
- Followed by two reminders at 1–2-week intervals.
- Results are publicly reported aggregated at whole trust level.
- Organisations are identified as within, above or below the statistical normal range.
- 136 000 patients annually administered AIPS.
- Response rate 50.3%.

We concentrated on patient experience questions, which explore detailed specific events, emotions and impressions that a patient encounters during a healthcare episode, and we did not include satisfaction questions. This allowed analysis of the organisational impact on granular, discrete elements of care. Patient experience questions are particularly valuable for guiding improvement as the information they return is detailed and specific. In order to limit the number of primary analyses, we made a priori selection of the two Picker core domains that included only one questionnaire item; (1) feeling treated with respect and dignity and (2) pain control.^18^ We selected these single item domains because domains and the questions within domains had been selected during survey design on the basis of patient priorities, and we therefore regarded questions that were in domains as most important. We used single item domains because multilevel regression methods only apply to logistic models and require binary data. While all questions in the AIPS have a range of available responses, single questions can be collapsed to binary data suitable for logistic analysis using the established Picker ‘problem/no problem’ matrix.^19^ All other Picker core domains consisted several items, and their aggregate scores had distributions that were censored at the maximal value, could not be converted to data suitable for regression analysis and could not be rendered into binary data suitable for multilevel modelling using any existing validated approach.

In order to determine if the selected primary analyses of respect and dignity score and pain control score reflected the broader behaviour of the survey, we performed secondary multilevel logistic analysis of the remaining individual questions that are included in the core Picker domains. We examined the correlation between respect and dignity score and pain control score and other questions, using Cronbach's α.

Changes in patient characteristics over time in each trust were investigated using regression models with age, gender, long-term conditions and disabilities as dependents, and trust and year as explanatory variables, in order to determine whether local case mix was stable, which would indicate that local data could be followed over time for improvement purposes without case mix adjustment.

Associations between patient characteristics and primary outcome experience scores, being treated with respect and dignity and pain control, were examined using Fisher's exact test.

We generated multilevel logistic regression models with random intercepts to evaluate the impact of patients and trust factors on each dependent variable using MLWin V.2.31 software (Rasbash J, Charlton C, Browne W, *et al*. MLwiN, Version 2.31. Centre for Multilevel Modelling, University of Bristol). We used reweighted iterative least squares and penalised quasi-likelihood method with outputs fed into a Markov chain Monte-Carlo model with 10 000 iterations which resulted in convergence for all analyses (Browne WJ. MCMC estimation in MLwiN v2.31. Centre for Multilevel Modelling, University of Bristol). While the data came from entire set of acute trusts in England, we regarded these as being sampled from a hypothesised super-population and modelled random effects at this level. We calculated partitioned variance between patient and AMU levels using a latent variable approach, on the basis that quality of experience expressed as Picker problem/no-problem scores can be regarded as a continuous variable collapsed to categorical data.^20^ Hierarchical levels were trust and patient. Independent variables were included on the basis of previous studies that had shown significant effects: age, gender, length of stay and long-term conditions.^8^ ^21^–^25^ We also included presence and impact of disability which is reported in the AIPS. We included size and teaching status, as defined by the English Department of Health, as trust level characteristics.^26^ ^27^

## Results

In the three-year period of study, AIPS questionnaires were administered after discharge to 413 677 unselected hospital inpatients in 167 trusts and 208 280 surveys were returned and useable. There were 8753 short stay emergency medical admissions that met our criteria from 142 trusts. Questions on long-term conditions and disabilities were completed by 7776 patients (88.8%). Demographic characteristics of included patients are shown in table 1, with data for patients excluded from analysis because of incomplete responses to questions on long-term conditions and disabilities.

**Table 1:** Demographic characteristics of included patients, and, for comparison, of patients excluded because of incomplete response to the conditions and disability section of survey

	All long-term condition disability questions complete (patients included) (n=7776: 89.1%)	All long-term and disability questions not complete (patients excluded) (n=977: 10.9%)
Age group		
16–35 years	8.0%	5.6%
36–50 years	15.2%	11.8%
51–65 years	27.9%	20.8%
66–80 years	44.1%	55.4%
>80 years	4.7%	6.3%
LOS 1 night	74.3%	74.1%
LOS 2 nights	25.7%	25.9%
Female	52.1%	57.3%
Male	47.9%	42.7%
Survey year 2008	33.0%	33.0%
2009	33.3%	31.4%
2010	33.7%	35.6%

LOS, length of stay.

Of questionnaires returned by patients who met inclusion criteria (n=8753), the majority (n=7782, 88.9%) included complete self-reported information on long-term conditions and disabilities. These are independent characteristics of individual patients, which cannot be meaningfully imputed from other questionnaire data, and we therefore used list-wise deletion of those with missing data (n=977, 11.1%).

Hierarchical modelling with partitioning of overall variance between trust level and patient level in a model that included patient age, gender and length of stay and responses to questions on whether the patients had a range of long-term conditions and disabilities, as well as trust level characteristics (size and teaching status), revealed that most of the variance in patient experience data are accounted for by patient level factors, with only a small proportion of overall variance attributable at the level of individual trusts (table 2). Our primary analyses of scores for ‘respect and dignity ‘and ‘pain control’ showed that 99.5% and 99.6%, respectively, of variance is explainable by patient level factors. Results for other patient experience questions that are constituents of the core Picker Domains, and which were included in this study as sensitivity analysis to confirm that the two items included as primary analyses are not behaving atypically in this survey, showed similarly that most variation is explainable at patient level, with results ranging from 91.7% of variance accountable to patient level for clarity of answers from doctors to 99.4% for amount of information given and for involvement in decisions (table 2).

**Table 2:** The patient experience questions used in this study and criteria for problem/no problem scores

Question	‘No problem’ responses; scored as 1	‘Problem’ responses scored as 0	Proportion of variance attributable to trust level	Mean (range) trust scores
Overall, did you feel you were treated with respect and dignity while you were in the hospital?*	Yes-always.	Yes-sometimes; no.	0.5%	0.75 (0.58–0.86)
Do you think the hospital staff did everything they could to help control your pain?*	Yes-definitely.	Yes-to some extent; no.	0.4%	0.65 (0.45–0.87)
In your opinion, how clean was the hospital room or ward that you were in?	Very clean; fairly clean	Not very clean; not clean at all.	4.6%	0.94 (0.79–0.99)
How clean were the toilets and bathrooms? that you used in hospital?	Very clean; fairly clean	Not very clean; not clean at all.	4.9%	0.90 (0.72–0.97)
When you had important questions to ask a doctor, did you get answers that you could understand?	Always	Sometimes; no	8.3%	0.62 (0.45–0.77)
Did you have confidence and trust in the doctors treating you?	Always	Sometimes; no	1.2%	0.73 (0.58–0.90)
When you had important questions to ask a nurse, did you get answers that you could understand?	Always	Sometimes; no	1.8%	0.62 (0.37–0.84)
Did you have confidence and trust in the nurses treating you?	Always	Sometimes; no	1.8%	0.72 (0.50–0.86)
Were you involved as much as you wanted to be in decisions about your care and treatment?	Definitely	To some extent; no	0.6%	0.46 (0.32–0.59)
How much information about your condition or treatment was given to you?	The right amount	Not enough; too much	0.6%	0.72 (0.60–0.89)
How would you rate how well the doctors and nurses worked together?	Excellent; very good; good.	Fair; poor	2.9%	0.91 (0.80–0.98)

*Items regarded as primary analyses.

Analysis was performed to examine whether the primary parameters that we had selected, respect and dignity and pain control scores, behaved similar to other Picker domain questions. We calculated Cronbach's α coefficients of 0.83 and 0.82, respectively, indicating correlation between individual patients' scores for the two questions we selected for primary analysis and the remaining Picker domain questions, and supporting the use of the selected questions to represent the behaviour of the broader AIPS.

Gender and age had significant effects on experience scores for feeling treated with respect and dignity and for pain control, with younger age and female gender associated with worse reported experience. Overall, 80.1% of men and 71.9% of women reported that they felt they were treated with respect and dignity (Fisher's exact test, p<0.0001), and 69.9% of men and 62.0% (p<0.0001) of women felt that staff did all they could to control pain. Among patients over 65 years of age, 82% felt treated with respect and dignity, against 69% of patients aged 65 years and under (p<0.0001), and 70% of patients over 65 felt staff did all they could to control pain, against 62% of patients aged 65 or less (p<0.0001). Self-reported patient level characteristics that were significantly associated with lower scores for feeling treated with respect and dignity were the presence of a long-standing physical condition (71.9% and 77.8% of patients with and without a long-standing physical condition feeling treated with respect and dignity, p<0.0001) and difficulties that were due to long-term conditions with everyday activities, with reading or writing, with peoples’ attitudes towards the patient, and with communication (71.3% and 80.5%, respectively, of patients with at least one reported disability vs no disability felt treated with respect and dignity, p<0.0001). Lower pain control scores were associated with long-standing physical condition (59.7% vs 69.3%, p<0.0001), and difficulties with reading, attitudes and communication (61.3% of patients with at least one disability and 71.6% of patients with no disability felt staff did all they could to control pain, p<0.0001). Trust teaching status and size had no statistically significant association with scores for any of the questions in Picker domains (table 3).

**Table 3:** β coefficient, SE and p value for patient and trust level factors; reference values in parentheses

	Respect and dignity			Pain control		
	β	SE	p Value	β	SE	p Value
Length of stay 2 nights (1 night)	0.042	0.061	0.49	0.015	0.078	0.84
Deafness or severe hearing impairment	0.025	0.150	0.86	0.037	0.120	0.75
Blindness or partially sighted	−0.037	0.150	0.80	0.221	0.190	0.24
A long-standing physical condition	−0.152	0.078	0.05	−0.278	0.095	<0.01
A learning disability	−0.023	0.214	0.91	0.385	0.300	0.20
A mental health condition	−0.162	0.117	0.16	−0.198	0.151	0.19
A long-standing illness, such as cancer, HIV, diabetes, chronic heart disease and epilepsy	−0.031	0.069	0.91	−0.071	0.065	0.28
Everyday activities that people your age can usually do	−0.218	0.083	<0.01	−0.136	0.101	0.18
At work, in education or training	0.224	0.157	0.10	0.269	0.179	0.09
Access to buildings, streets or vehicles	−0.159	0.087	0.07	−0.101	0.107	0.34
Reading or writing	−0.256	0.127	0.04	−0.519	0.162	<0.01
People's attitudes to you because of your condition	−0.531	0.109	<0.01	−0.357	0.151	0.02
Communicating, mixing with others or socialising	−0.214	0.098	0.03	−0.307	0.127	0.02
Age 36–50 (16–35)	0.363	0.095	<0.01	0.174	0.123	<0.01
Age 51–65 (16–35)	0.927	0.091	<0.01	0.784	0.119	<0.01
Age 66–80 (16–35)	1.377	0.095	<0.01	0.829	0.121	<0.01
Age >80 (16–35)	1.265	0.164	<0.01	0.929	0.215	<0.01
Female (male)	−0.464	0.059	<0.01	−0.261	0.070	<0.01
Medium size trust (small)	−0.016	0.074	0.83	0.165	0.089	0.06
Large size trust (small)	−0.012	0.079	0.88	0.125	0.094	0.18
Teaching (non-teaching)	0.023	0.086	0.79	−0.055	0.109	0.61

For long-term conditions and disabilities, reference value is ‘not present’.

There was considerable variation between trusts in the proportion of patients reporting long-term conditions and disabilities (table 4). There was no significant year-to-year difference for proportions of patients with long-term conditions or disabilities in each individual trust, or for gender or age distributions.

**Table 4:** Survey questions on self-reported long-term conditions and resulting disabilities that were included in this study as independent variables

Survey questions on the presence of long-term conditions	Mean percentage of patients per trust answering ‘yes’ (range)
Do you have any of the following long-standing conditions?	
Deafness or severe hearing impairment	14 (5–23)
Blindness or partially sighted	5 (0–13)
A long-standing physical condition	31 (18–48)
A learning disability	1 (0–6)
A mental health condition	6 (0–16)
A long-standing illness, such as cancer, HIV, diabetes, chronic heart disease and epilepsy	35 (21–50)
I have no long-term conditions	34 (21–50)
Survey questions on disabilities due to long-term conditions	Mean percentage of patients per trust with at least one long-term condition answering ‘yes’ (range)
Does this condition(s) cause you difficulty with any of the following?	
Everyday activities that people your age can usually do	40 (19–48)
At work, in education or training	9 (2–20)
Access to buildings, streets or vehicles	17 (0–28)
Reading or writing	8 (0–15)
People's attitudes to you because of your condition	8 (2–18)
Communicating, mixing with others or socialising	13 (3–25)
No difficulties	44 (28–69)

Percentage of patients reporting long-term conditions in individual trusts are shown as median (range). Values for disabilities resulting from conditions represent percentages of patients with at least one long-term condition who report each disability.

### Missingness analysis

Several of the inclusion criteria depended on self-reported survey responses (stay on initial ward, admission to enhanced care area, emergency vs elective admission) so it is not possible to comment on the number of patients who met the inclusion criteria but did not return a questionnaire. Patients who returned forms and did meet inclusion criteria but did not complete the long-term conditions and disability section of the survey were excluded. Excluded patients were similar in terms of gender and length of stay, but there was a slight difference in age distribution, with slightly more patients over 66 years not completing the long-term condition and disability section (table 1).

## Discussion

This is the first study to the best of our knowledge that has used multilevel hierarchical analysis to apportion the variation in patient experience scores between trust and patient levels for single, common, reproducible microsystem in each organisation and that has used data for all providers in an entire country. Previous multilevel analyses of inpatient experience scores for a range of items have shown the proportion of variance attributable at organisation level of only 0.8–5.3% and 0.1–5.4% in two studies in the Netherlands, and 2–6% for 83 hospitals in Canada.^6^^–^8 A similar phenomenon has been seen at a suborganisational level with only 0.9–2.1% of variance of inpatient experience explained by specialty.^8^ ^9^ We had hypothesised that the quality of experience delivered by a stereotyped reproducible microsystem, the acute medical admission process, might show more local trust level contribution to variance. However, our findings did not support this, with results that were similar to those of other studies. Patient level factors, including demographics and self-reported disability and impairment, explained the majority of the variance in experience scores, with only a small contribution to the variance from either trust identity or trust level factors (size and teaching hospital status).

We selected two survey items a priori to be primary outcome parameters so as to avoid multiple analyses. These two items, feeling treated with respect and dignity and perception of staff's efforts to control pain, revealed a particularly low degree of organisational influence. However, other questions included in Picker domains also showed low organisational influence. The highest value for organisational influence as a proportion of total variance was for the question on ‘getting answers you could understand from doctors’. It is difficult to appreciate why clarity of communication would be consistent across a trust, although geographical differences in patient educational level could explain the organisational influence for this item. Cleanliness of toilets and bathrooms also showed more variation attributable to trust than other items, and this could be related to resourcing and commissioning of trust-wide facility services.

A possible explanation for the finding of weak trust level influence on experience would be that generic surveys such as the AIPS, designed on the basis of research involving the entire inpatient population, do not include questions that reflect the specific needs of patients in any one particular microsystem, or alternatively, questions that focus on the aspects of service that are sensitive to organisational influence. Further research into the needs, wishes and priorities of subgroups of patients may lead to development of a bank of more meaningful, microsystem-specific surveys. Furthermore, this could include a deliberate selection of questions that are sensitive interorganisational discriminators, so that future survey results would be more suitable for external comparison. Future research should include more comprehensive inquiry into patient characteristics, such as educational attainment, which has been shown to be associated with experience scoring, as well as other exploratory items such as income and indices of deprivation to explore equity.^8^

An alternative explanation would be that perceived/reported experience is affected by patient characteristics to such an extent that trust level influence is masked. This could be because subgroups of patients receive different quality of care, or perceive the same care differently, or are predisposed to response more positively or negatively to surveys (or because of reverse bias, with patients who recall a poor experience being more likely to report long-term conditions and disabilities due to affective spill over). Without knowing how much reporting bias present, it is difficult to know whether case mix adjustment should be applied before meaningful external comparisons can be made. On the one hand, adjustment for patient factors might amplify trust level influence on scores. However, the danger would be that real differences in experience for subgroups are masked, and the fact that certain groups receive worse treatment becomes invisible in the adjusted output.

Local data can be used internally to inspire and guide improvement, without recourse to comparison with other organisations' results.^28–31^ When used in this way, adjustment for case mix may not be relevant. As shown in our analysis the mix of patient characteristics in single centres remains stable, at least over the 3 years studied and this time period would exceed the life of many improvement initiatives. Local shortfalls in specific scores can be identified by comparison with what would be desirable performance rather than by benchmarking against other hospitals. For example, the finding that 35% of patients who had pain did not feel staff did all they could to treat the pain invites attention, regardless of whether other trusts are doing better or worse.

Our findings prompt an important question: what hierarchical level within an acute provider organisation, if any, delivers consistent experience, and what levels, if any, are subject to corporate influence, with respect to delivery of good experience? Our findings suggest, at least in acute medicine, and taking into account the limitation of using a generically developed survey, that this level sits below the Acute Admission Unit. However, below that level there are unlikely to be teams of doctors or nurses that retain their membership over time, because of the complexity of shift patterns. This raises the possibility that the highest level of consistent experience quality could be the individual member of staff. If this is the case, the mixture of effects of the large number of staff in a clinical unit may produce an average that is similar in every trust, explaining the lack of organisational impact on scores. It may be the case that there is currently no pervasive organisational level influence on front line patient-centred behaviours. Trusts are large organisations with thousands of employees and parallel authority structures based around disciplines, rather than a simple pyramid of control. The structure of the oragnisational networks of influence in hospitals has not been mapped, it is not currently known whether the connections between trust strategy and patient facing staff are adequate to transmit consistent influence or even whether these connections exist at all. That is not to say that this must remain the case, and trust-wide quality initiatives may generate better networks and enhance links between senior leadership and microsystems. It is interesting to speculate that well-conducted improvement projects in a subset of trusts may lead to a situation where surveys are better able to discriminate between organisations because of a widening performance gap.

## Conclusion

The AIPS, a generic patient experience survey developed for the use across the hospital inpatient population, reveals only limited organisational influence on scores when a stereotyped microsystem, the acute medical admission service, is isolated. This may be because a generic survey does not include questions that link to the specific needs of patients in a particular microsystem, or questions that are good at discriminating between trusts. Further research would be useful to develop subspecialty experience surveys with better performance. Alternatively, it is possible that trusts lack mechanisms to disseminate influence over patient-centred behaviours to the clinical front line. This should prompt research into how influence flows within hospital, and how this can be performed more effectively. If the discrimination between organisations cannot be increased, either through use of different surveys or through more effective central control of patient centredness, the value of collecting and publishing national data for comparison falls into question, and experience scores generated locally would be best used to drive and monitor local improvement.
